# Ministring DNA (msDNA): a novel linear covalently-closed DNA with enhanced stability for gene and cell therapy applications

**DOI:** 10.1038/s41598-025-98730-5

**Published:** 2025-05-02

**Authors:** Wai Kuen Hung, Siddarth Chandrasekaran, Olga Zaslaver, Ming Zhu, Jamie Lam, Steven Hersch, Peyman Mokarami, Roderick A. Slavcev, Nafiseh Nafissi

**Affiliations:** 1 Mediphage Bioceuticals, 661 University Avenue, Suite 1300, Toronto, ON M5G 0B7 Canada; 2 School of Pharmacy, University of Waterloo, 10A Victoria St S, Kitchener, N2G 1C5 Canada

**Keywords:** Keywords, Ministring DNA, Linear covalently closed DNA, Gene therapy, Chemical and mechanical stability, Lyophilization, Biotechnology, Drug discovery, Molecular biology

## Abstract

The quality and fidelity of DNA vectors used in genetic medicine and gene therapy either as starting material for manufacturing or as therapeutic ingredients play a critical role in determining ultimate clinical success. Ministring DNA (msDNA), is a novel minivector that is a linear covalently-closed (LCC) double-stranded DNA molecule devoid of immunogenic bacterial sequences (e.g., origin of replication, antibiotic resistant cassette). Similar to traditional plasmids, msDNA is manufactured in vivo in *E. coli* and therefore benefits from the scalability of *E. coli* -based systems and the ~ 1000-fold enhanced fidelity conferred by the mismatch repair (MMR) mechanism. In this paper, we address the improved stability of msDNA. We show that due to the torsion-free structure, msDNA is more stable to chemical and mechanical stress than conventional plasmid DNA. Moreover, we demonstrate that lyophilization can further improve the long-term stability of msDNA, reducing the need for cold chain storage. Therefore, we propose that msDNA can be a new paradigm for genetic medicine by offering genetic material with lower immunogenicity, reduced risk of insertional mutagenesis, and higher fidelity and stability.

**Supplementary Information:**

The online version contains supplementary material available at 10.1038/s41598-025-98730-5.

## Introduction

Gene therapy has experienced remarkable advancements in recent decades, driven by significant innovations in vector design, molecular biology, and clinical applications. This rapid evolution gained further momentum during the COVID-19 pandemic, the utilization of mRNA-LNP based vaccines to immunize the world against the pandemic highlighted the potential of nucleic acid-based vaccines. Gene therapy involves a range of modalities including viral vectors such as adeno-associated viruses^1^ (AAVs) and lentiviral vectors^2^ (LVs), oligonucleotides^3,4^, RNA^5^, and DNA-based therapies^6,7^. Each modality presents distinct advantages and limitations which are influenced by factors such as target tissue, gene expression levels/longevity, and immune response. Notably, DNA vectors, particularly plasmid DNA, are pivotal components in these therapies, serving either as active pharmaceutical ingredients (APIs) or as starting material for producing viral vectors and RNA. Consequently, there is a pressing need to develop high-quality DNA-based vectors^8,9^.

DNA vectors are extremely stable due to their double-stranded nature^10,11^ and have distinct advantages when used as APIs. DNA molecules are composed of a stable sugar-phosphate backbone that is further stabilized by extensive hydrogen bonding between DNA bases on opposing strands^12^. This stability endows them with specific advantages as APIs in both gene supplementation/addition and gene editing platforms. Furthermore, this stability extends to 4 °C and room temperature, allowing for storage and shipment without significant cold-chain storage. Room temperature stability is a challenge for RNA-based technologies^7,13^, which typically require sub − 20 °C to -80 °C for long-term storage, a fact particularly highlighted during the COVID-19 pandemic.

The utilization of stable, fully closed DNA vectors—free from open or nicked ends—is crucial across multiple applications, from viral vector manufacturing to their role as APIs in gene and cell therapies^14^. In viral vector production, DNA enables efficient amplification and uniform packaging, thereby reducing the risk of generating incomplete or non-functional particles^15^. Likewise, high-quality DNA templates lead to cleaner transcription and higher yields of full-length mRNA, which is vital for the potency and safety of mRNA-based vaccines and therapeutics^16^. Conversely, DNA exhibiting nicked ends or structural instability can adversely affect both manufacturing processes and clinical outcomes, as open or unstable DNA is more susceptible to degradation by nucleases, compromising yields and leading to improper viral packaging or truncated mRNA transcripts. Such degradation diminishes the quality of the therapeutic product and raises safety concerns. Instability in DNA vectors used for clinical applications, such as gene therapy or cell therapy, can lead to rapid degradation or unwanted immune responses, undermining gene delivery efficacy and resulting in incomplete therapeutic effects^17^. In gene editing, unstable DNA sequences can introduce off-target mutations or incomplete edits, jeopardizing therapeutic precision and safety. Therefore, ensuring DNA integrity is paramount for achieving effective and safe therapeutic outcomes.

The most popular choice for DNA vectors is plasmid DNA, which has gained popularity due to its relative ease of manipulation and production in *E. coli*. Plasmid DNA has been well-studied and used at the industrial scale with significant improvements in both upstream and downstream manufacturing^18,19^. However, plasmids have not been widely used as APIs for two main reasons: (1) high immunogenicity and (2) insertional mutagenesis. Plasmid DNA contains prokaryotic DNA sequences such as immunostimulatory CpG motifs, bacterial origins of replication (ori), and antibiotic resistance cassettes, which are necessary for bacterial replication but can be severely immunogenic. To overcome these challenges, mini-DNA vectors, such as nanoplasmids^8,20^ or minicircles^21,22^, were developed to minimize or eliminate these immunogenic DNA sequences. While these mini vectors are not immunogenic, they still pose the potential risk of insertional mutagenesis and unregulated vector integration^23^.

**Fig. 1:**
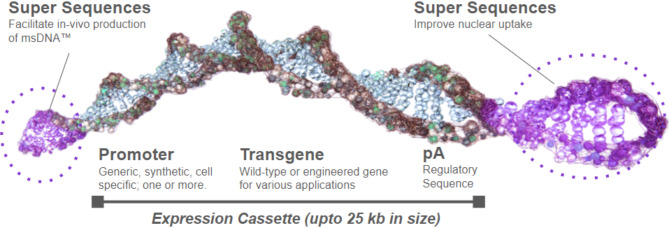
The msDNA is a linear covalently-closed double-stranded DNA vector free of bacterial backbone DNA. msDNA carries a minimal expression cassette comprised of the eukaryotic promoter, transgene or genes of interest, and regulatory sequences. The ends of the msDNA are covalently closed by a protelomerase induced hairpin formation. [Image obtained with permission from Mediphage Bioceuticals Inc.]

To further address these disadvantages, linear covalently-closed vectors (LCC vectors), such as doggybone DNA^24^ (dbDNA) or ministring DNA (msDNA)^23,25–27^, were developed. These LCC DNA vectors are devoid of bacterial backbone and feature a linear structure containing only the necessary genetic elements (Fig. 1), such as the gene of interest (GOI), promoter, and enhancer—for gene expression. Additionally, LCC mini vectors mitigate the risk of insertional mutagenesis by creating double-stranded DNA breaks that can result in cell death in an inadvertent integration into the host genome^23,27^. Recent advancements in cell-free technologies have driven a movement toward the synthetic production of DNA by polymerase chain reaction (PCR) or rolling circle amplification (RCA). Numerous studies have highlighted the impact of the mismatch repair (MMR) and proofreading mechanism in fixing potential mutations during replication in *E. coli*^28,29^. Our recent findings^30^ have corroborated these observations, we have demonstrated that DNA manufactured in vivo in *E. coli* (such as plasmid or msDNA) has 80- to 3000-fold higher fidelity^30^ compared to in vitro and PCR-based manufactured DNA due to the MMR mechanism of *E. coli*-based manufacturing^28,29^.

As a result, msDNA (Fig. 1) emerges as a promising candidate not only as a starting material but also as an API for gene and cell therapy applications. The advantages of msDNA include lower immunogenicity due to the absence of a bacterial backbone^23^, reduced risk of insertional mutagenesis^26^ linked to its linear covalently-closed structure, and enhanced fidelity stemming from the MMR system in *E. coli*^30^. The msDNA construct comprises solely the expression cassette, encompassing the promoter, transgene, and regulatory elements, all flanked by two supersequences (SSeq™) equipped with protelomerase recognition sites and nuclear uptake enhancer elements. These protelomerase sites facilitate in vivo manufacturing of msDNA in cells expressing the designated site-specific protelomerase^26,27^. Additionally, since msDNA is produced in *E. coli* and utilizes similar manufacturing processes as plasmid DNA, it benefits from the same scalability and manufacturing advantages afforded to plasmid DNA manufacturing, and unlike other LCC mini-DNA constructs, enhanced fidelity resulting from the mismatch repair (MMR) system in *E. coli*.

In this work, we aimed to investigate the stability of msDNA, particularly if the linear covalently-closed and double-stranded nature of the DNA provides any additional or enhanced stability. First, we studied the stability of msDNA to chemical and mechanical stress that are typically encountered by DNA during the formulation and encapsulation processes^31–34^, as well as during ex-vivo gene delivery, particularly for cell therapy and gene editing applications. Multiple delivery methods exist for DNA, including cationic lipids, ionizable lipids, and polymer nanoparticles, each with distinct advantages and limitations. In this paper we focus on the process parameters typically encountered for ionizable lipids as they represent the current state-of-the-art due to their lower toxicity and higher efficacy^35,36^.

Second, we developed a lyophilization method^37^ to improve the long-term stability of msDNA and evaluate its universality across various msDNA constructs. Lyophilization offers a promising approach to enhance DNA stability; however, the dehydration inherent to this process can disrupt the DNA hydration sphere, potentially leading to structural damage. Studies demonstrate that without protective agents, lyophilization significantly reduces transfection efficiency and biological activity^38^. While the effects of lyophilization on plasmid DNA^39^ (pDNA) have been explored, its impact on linear covalently closed DNA, such as msDNA, remains underexamined.

## Methods

### Agarose gel electrophoresis (AGE)

1% Agarose gels were made in Tris-acetate-EDTA (TAE) buffer containing 1X GelRed Nucleic Acid Stain. Electrophoresis was performed in the BIO-RAD Horizontal Electrophoresis system at 120 V for 30–60 min. Images were generated by UV transillumination using the BIO-RAD ChemiDoc Imaging System and quantified using Fiji-ImageJ software v1.54-f. NEB 1 kb Plus DNA ladder (Figure S3) was used for all gels in this study. A linear background correction was applied to all the peaks before quantification. AGE analysis has an inherent ~ 5–10% error due to sample loading and data processing variability; thus, recoveries of 95–105% are interpreted as complete recovery. For pDNA containing multiple isoforms, only the supercoiled fraction was quantified, as it is the primary form used in therapeutic contexts.

### Preliminary stability measurements

msDNA was stored at 35 ng/µL in aqueous solutions at various temperatures (4 °C, room temperature, −20 °C, −80 °C) in centrifuge tubes. Samples were then analyzed at defined time points (1 week, 1 month, 3 months, and 6 months) for degradation using either AGE or Ultra-high-performance liquid chromatography (UHPLC). UHPLC measurements were performed on a Thermo Scientific™ Vanquish™ Flex UHPLC system using a 0.3 mL anion exchange column. Approximately 100–300 ng of msDNA was loaded onto the column, and a linear salt gradient (0–700 mM NaCl) buffered by 100 mM Tris (pH 8.0) was used to elute the msDNA from the column. Purified msDNA eluted as a single peak at a defined elution time determined by the size and nature of the DNA.

### Mechanical stress

Weak sonication (30–45 watts/liter) was performed at room temperature with 35 kHz power generated by the VWR Ultrasonic cleaner. 40 µL of 50 ng/µL pDNA and msDNA samples were aliquoted into 1.7 mL microcentrifuge tubes and sonicated for 5 s, 10 s, 30 s, 1 min, and 10 min. Strong sonication was performed using the Branson Digital Sonifier SFX 550 with a 1/8 inch diameter tapered probe at 20% amplitude (~ 100,000 watts/liter) on a 0 °C ice bath. 1 mL of 10 µg/mL plasmid DNA and msDNA samples were sonicated for 5 s, 10 s, 30 s, and 1 min with a 1 s operation time and 3 s rest time. After sonication, the samples were directly loaded onto a 1% agarose gel for further analysis.

### Chemical stress

20 µg of pDNA and msDNA were incubated in various 10 mM buffers (10 mM Acetic Acid-NaOH for pH 3 and pH 5; 10 mM Tris HCl for pH 7 and pH 9; and 10 mM Sodium Bicarbonate for pH 10.5) in a 40 µL final volume for 1 h or overnight (~ 16 h). The incubation reaction was stopped by a ~ 33-fold dilution into 1X CutSmart Buffer (NEB, pH 7.9). These samples were incubated with T5-exonuclease (NEB) for 30 min at 37 °C. The T5-exonuclease reaction was quenched by adding 1X gel loading dye (containing 10 mM EDTA) and the samples were loaded onto a 1% agarose gel for analysis. Samples were treated with T5-exo, chosen for its ability to process damaged DNA species^40^, to detect stability loss not detectable by AGE alone.

### Lyophilization

Lyophilization was performed using a LABCONCO Benchtop Freeze Dryer. All samples were dissolved in H_2_O or in 10 mM Trehalose aqueous solution and stored in 2 mL microcentrifuge tubes at −80 °C for 2 to 3 h until they were completely frozen. The samples were placed into the freeze-dryer and a vacuum was applied overnight (~ 16 h) to completely lyophilize the samples. The lyophilized samples were reconstituted in the same volume of water and further analysis was carried out. UV-Vis measurements were carried out using a 2 µl sample on a nanodrop (Thermo Scientific™ NanoDrop™). UV-Vis spectra were recorded using a Nanodrop with variable pathlengths, normalized to a 10 mm pathlength, yielding absorbance values up to 20, corresponding to a DNA concentration of ~ 1 µg/µL. DNA damage was measured by AGE after incubation with T5-exonuclease for 30 min at 37 °C.

### Statistical analysis

A one-way analysis of variance or t-test was carried out to test whether any change in process or treatment results in significantly different recovery of DNA. The analysis was carried out using the t.test or anova function in R (Version 4.4.2, {stats} package) and summary() function was used to extract parameters which are shown in the relevant tables (Table S2 and S3) in the supplement. In the case of ANOVA model results were used for a *post hoc* Tukey “honestly significant difference” test to identify the pairs of comparisons with significantly different means using the TukeyHSD() function.

### Cell culture

Transfection of DNA samples (before and after lyophilization) was performed in Human Embryonic Kidney cells- HEK293 cells (ATCC)- using the jetOPTIMUS transfection reagent (Sartorius) to evaluate green fluorescent protein (GFP) expression. Cells were seeded 24 h prior to transfection at a density of 100,000 cells/well in a 24-well plate. Samples were formulated at a 2.0:1.0 reagent (µL) to DNA (µg) ratio, with a total of 0.25 pmol total DNA per well. Transfection was performed with cells in transfection media (DMEM + 10% FBS), which was replaced with cell-growth media (DMEM + 10% FBS + 100 U/mL penicillin + 100 µg/mL streptomycin) 4 h after transfection. GFP expression was evaluated via flow cytometry (BD LSRFortessa Cell Analyzer) 2 days post-transfection.

### DNA manufacturing

Both plasmid DNA (pDNA) and msDNA were obtained from Mediphage Bioceuticals Inc. (Toronto, Canada). Several msDNA constructs were produced for this study, with salient features summarized in Table 1. The msDNA constructs ranged in size from 1.5 kb to 10.5 kb and exhibited a GC content between 42% and 62%, with applications spanning various sub-fields of gene therapy, including non-viral gene supplementation, starting material for manufacturing of lentiviral, RNA, and adeno-associated virus (AAV).

**Table 1:** Overview of the various msDNA constructs utilized in this study. For each construct, the table presents the following information: size (in kilobases), GC content, and specific genetic elements or features present. This comprehensive summary facilitates comparison and understanding of the constructs’ characteristics and their relevance to the study.

msDNA Construct	GC (%)	Size (kb)	Field of Use	Features
A*	59	4.3	Non-viral Gene Therapy	Bicistronic DNA GC-rich promoter with GFP and luciferase reporter gene
B	51	6.7	Lentivirus manufacturing	GFP reporter construct containing a long terminal repeat (LTR)
C	50	5.3	Non-viral Gene Therapy	Bicistronic DNA with tissue specific promoter expressing luciferase gene and a therapeutic hydrolase enzyme
D	62	4.9	AAV manufacturing	GC rich promoter with GFP reporter gene but flanked with inverted terminal repeats (ITRs)
E	53	10.3	AAV manufacturing	Large DNA construct encoding the AAV Helper gene
F	42	1.6	RNA manufacturing	Small construct containing a GFP gene with long polyA tail
G	52	3.7	Non-viral Gene Therapy	Bicistronic DNA with proprietary promoter, GFP and luciferase reporter genes
M	53	3.1	Non-viral Gene Therapy	Minimal luciferase reporter gene

*Note: msDNA lacks the bacterial backbone, no bacterial ori and antibiotic-resistant cassette. Therefore, the corresponding plasmid DNA (pDNA) is ~ 3.1 kb larger i.e., for msDNA construct A (4.3 kb), the corresponding precursor pDNA is ~ 7.4 kb with 55% GC content. Only pDNA for Construct A was used in this study.

## Results

### Preliminary results indicate that msDNA is stable in aqueous solution

DNA molecules, particularly double-stranded DNA (dsDNA), must maintain their structural integrity under various environmental stresses to remain stable. This enhanced structural integrity is crucial for the proper functioning of living organisms and for the preservation of genetic information. This stability is achieved by the extensive hydrogen bonding network between the two strands of DNA. msDNA, due to its double-stranded nature, benefits from enhanced stability compared to single-stranded nucleic acids such as mRNA. Further, its linear covalently closed (LCC) topology further distinguishes it from supercoiled dsDNA molecules. This LCC topology of msDNA renders it torsion-free^25^, which is hypothesized to improve its stability.

**Fig. 2:**
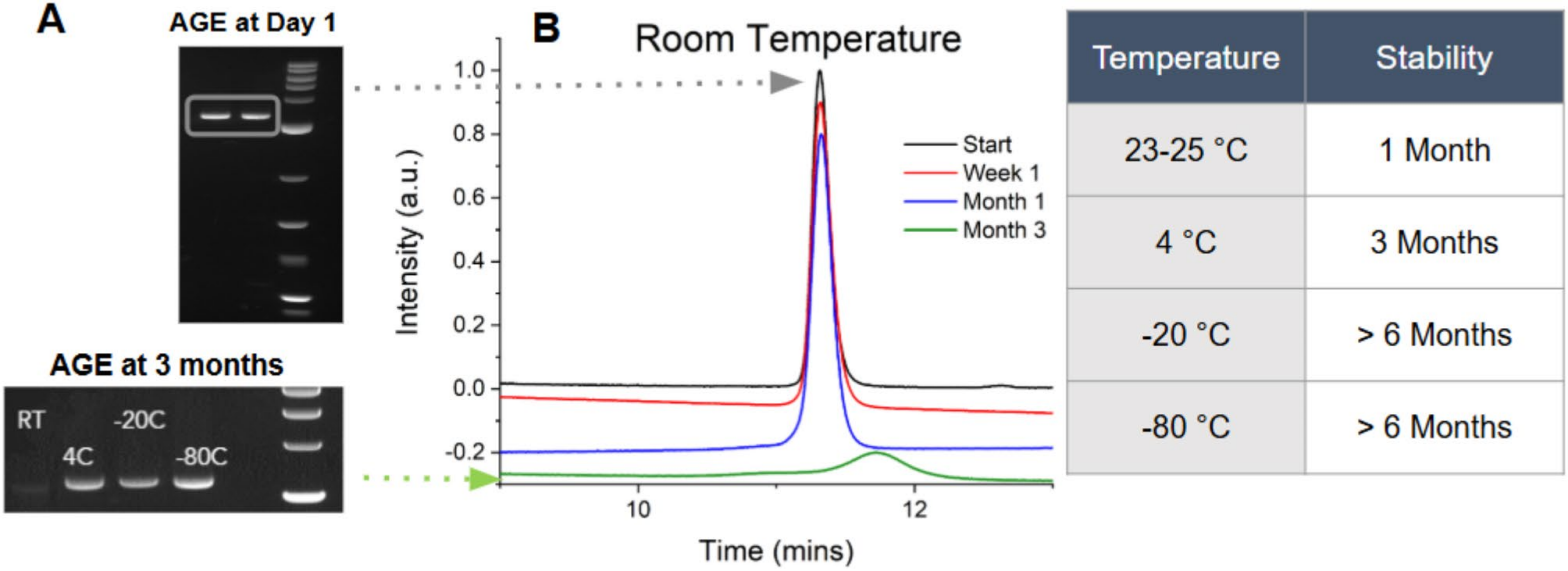
Preliminary stability test of msDNA (Construct M, 3.1 kb, 53% GC) in nuclease-free pharmaceutical grade water. (**A**) AGE of samples at the start of the experiment (Day 1) and after 3 months of storage, highlighting significant DNA degradation over time at room temperature (23–25 °C). All temperatures are in degrees Celsius (°C). (**B**) Ultra-high-performance liquid chromatography (UHPLC) traces of the sample at various time intervals (Start, 1 week, 1 month and 3 months). For detailed UHPLC traces at each time point, refer to Figure S1.

In our initial assessments, we conducted stability tests on msDNA using agarose gel electrophoresis (AGE) and ultra-high performance liquid chromatography (UHPLC) analysis (Fig. 2 and Figure S1). Our findings demonstrate that msDNA (Construct M) remains stable when stored at room temperature (in the absence of light) for a minimum of one month before degradation occurs. While AGE suggests a minor reduction in band intensity at 4 °C after 3 months, UHPLC analysis confirms no significant degradation, indicating stability is maintained. Further, when stored at 4 °C (Figure S1), degradation of msDNA (Construct M) is observed at the six-month mark, as evidenced by a noticeable shift in the UHPLC trace. This indicates that msDNA (Construct M) can be stored at 4 °C for a period of up to 3 months while maintaining stability. Construct M is a relatively small msDNA construct (3.1 kb) and may exhibit greater stability compared to larger msDNA constructs.

### MsDNA exhibits greater stability to mechanical stress compared to isogenic supercoiled plasmids (pDNA)

Next, we assessed the stability of msDNA to mechanical forces in the form of weak (30–45 watts/liter) and strong sonication (100,000 watts/liter) for short periods of time (< 1 min). These short periods of sonication are typically used during the API formulation process to homogenize the solution and reduce the particle size^41^. It is known that sonication can degrade DNA by breaking hydrogen bonds and causing strand cleavage, leading to fragmentation into smaller pieces-300–500 bp fragments^42^. We compared the stability of msDNA to traditional supercoiled plasmids to understand the relative stability of our linear double-stranded DNA molecule (msDNA). We hypothesized that msDNA would be more stable due to its linear torsion-free structure^25^.

**Fig. 3:**
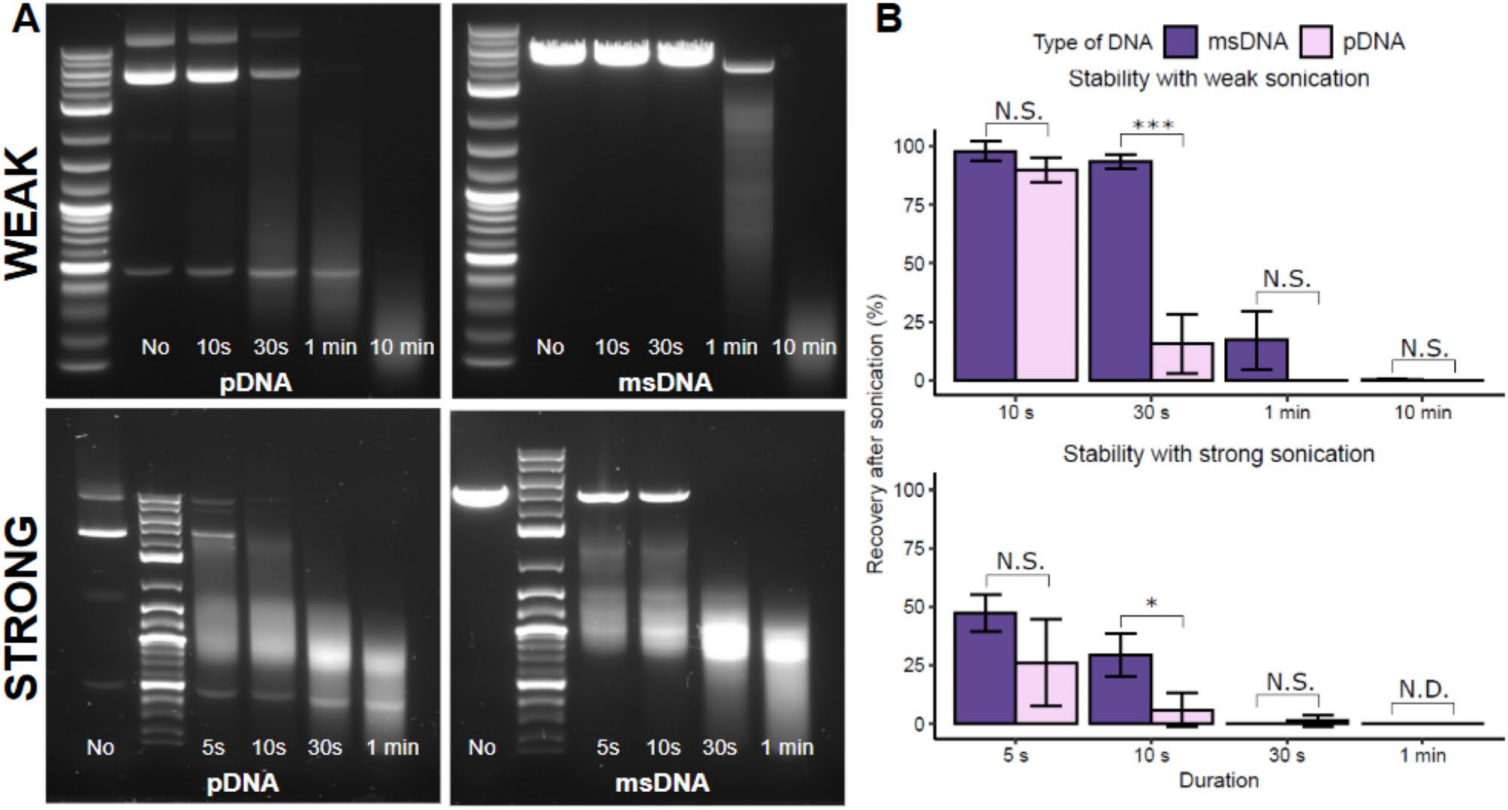
Stability assessment of msDNA (Construct A, 4.3 kb, 59% GC) and isogenic pDNA under mechanical stress. Both pDNA and msDNA were subjected to mechanical forces by weak (bath; 30–45 watts/liter) and strong (probe-tip; 100,000 watts/liter) sonication. The msDNA demonstrated stability under weak sonication and exhibited greater stability than pDNA under strong sonication for brief exposure times. (**A**): Agarose gel electrophoresis AGE of pDNA and msDNA following weak and strong sonication and (**B**): Quantitative analysis of the AGE comparing the stability of msDNA with pDNA (for pDNA only the supercoiled fraction is considered for quantification). Bars show the average of three biological replicates, and error bars show one standard deviation. T-test: ***p < 0.001, *p < 0.05, the statistics data is summarized in Table S2.

Our results (Fig. 3) indicate that both msDNA and pDNA remain stable after weak sonication in a water bath for a short period of time (i.e., ~ 10s at room temperature). However, msDNA appears to be stable after weak sonication for extended periods of time (~ 30s), while pDNA shows signs of degradation with approximately 50–75% DNA loss. When subjected to strong sonication, both msDNA and pDNA demonstrate marked damage, leading to nearly complete loss when sonicated for > 30 s. For shorter sonication times (5 and 10 s), msDNA retains ~ 45–47% and ~ 35 − 30% survival, respectively, compared to only ~ 25% and ~ 5% for pDNA. These results suggest that msDNA exhibits enhanced stability against mechanical forces.

### MsDNA exhibits greater stability to chemical stress compared to supercoiled plasmid (pDNA)

To further evaluate stability to various chemical stresses encountered during the formulation process^32^, msDNA and isogenic pDNA were incubated in various buffers with pH values ranging from 3 to 10.5 for either 1 h or overnight (16 h). DNA is known to undergo extensive alkaline denaturation above pH 11.5^43^, and significant depurination and damage below pH 3^44^. As DNA damage (such as nicking, partial denaturation) cannot be easily recognized on msDNA due to its already linear structure, we treated the incubated samples with T5-exonuclease (T5-exo) before running them on an agarose gel. T5-exo was chosen as it processes a wide range of damaged ^40,45^ DNA species such as single stranded-, nicked- and denatured- DNA species. If the DNA molecules are intact without any DNA damage, then no loss is observed in the samples exposed to T5-exonuclease.

**Fig. 4:**
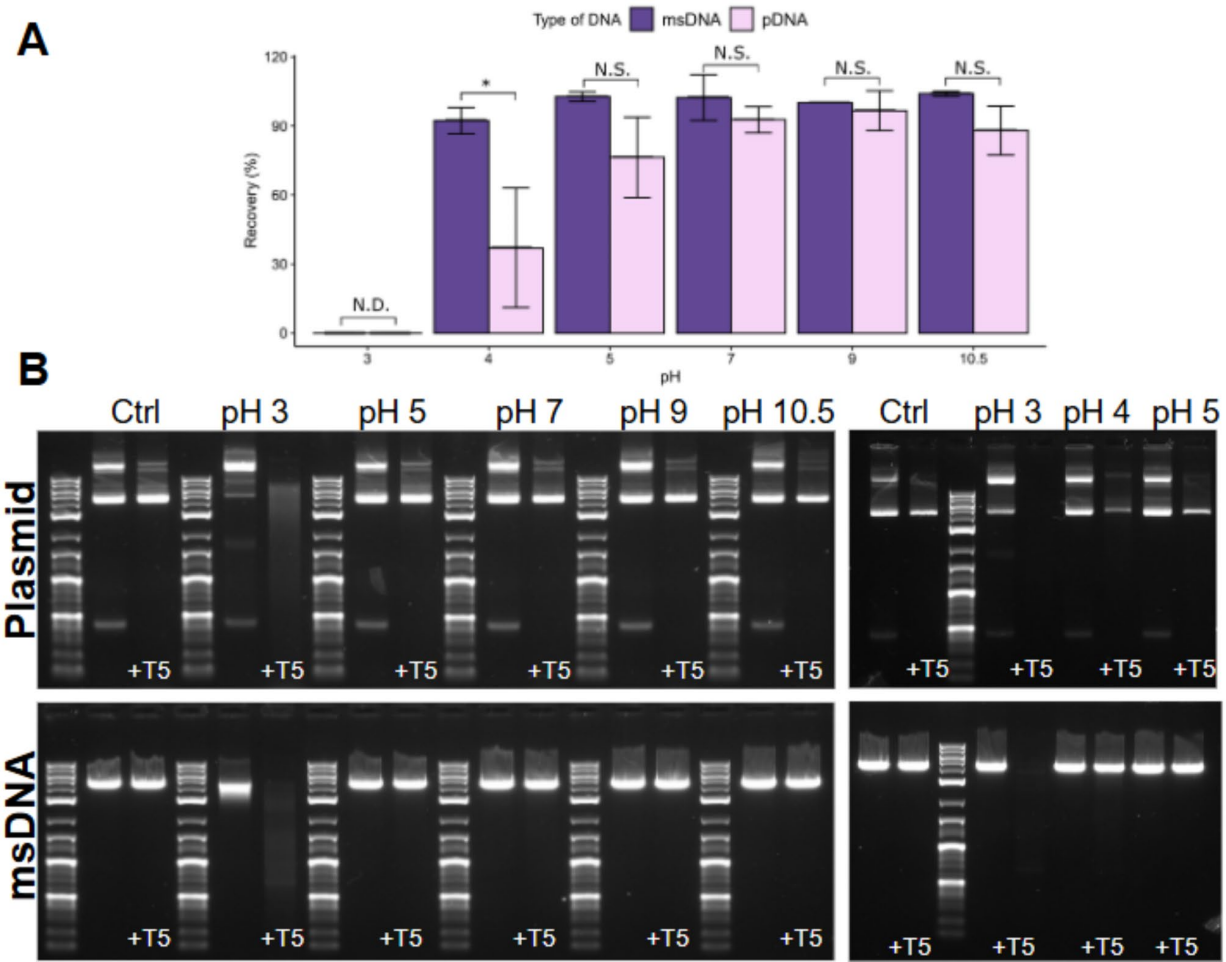
Stability assessment of msDNA (Construct A, 4.3 kb, 59% GC) and isogenic pDNA under chemical stress following overnight incubation. (**A**) Quantitative analysis of agarose gel electrophoresis comparing the stability of msDNA with pDNA (note: only the supercoiled fraction of the pDNA is considered for analysis). Bars show the average of three biological replicates, and error bars show one standard deviation. T- test: *p < 0.05,, the statistics data is summarized in Table S3. (**B**) AGE results of pDNA and msDNA at various pH conditions after approximately 16 h (overnight) of incubation. Both msDNA and pDNA exhibit stability within the pH range of 5 to 10.5 but show signs of degradation at pH 4 or below. Notably, msDNA remains stable at pH 4, while pDNA does not. Note: For pDNA, only the supercoiled fraction (the brightest band between 4–5 kb) is considered for quantification as the other isoforms (e.g., nicked, open circular, and linearized) present in pDNA are fully processed by T5.

Our results indicate that both msDNA and pDNA are stable between the pH range of 5-10.5 and unstable at pH 3 (Fig. 4, Figure S2). Notably, at pH 4, msDNA retained stability even after 16 h of incubation, while a substantial portion (~ 70% loss) of the supercoiled pDNA was damaged. This finding is particularly relevant for lipid nanoparticle (LNP) formulations composed of ionizable lipids, which are the current state-of-art for LNP formulations for clinical applications ^31,33,35,36,46^. For LNPs based on ionizable lipids, the DNA stock is often diluted in buffers at these low pH values. Typically for LNP applications the DNA is diluted in acetate buffers at pH 3 or 4 before mixing with lipids^31^. Overall, this enhanced stability of msDNA to chemical stress at pH 4 has the potential to significantly improve the quality of the DNA cargo as therapeutic ingredients for non-viral gene therapy applications.

### Improving msDNA stability by lyophilization

#### Preliminary tests

To enhance the stability of msDNA for long-term storage and to lower its degradation at room temperature, we conducted extensive lyophilization studies. The process of lyophilization offers the following main advantages: (1) reduced sample volume and weight, and (2) removal of the need for cold chain storage. Lyophilization has the potential to improve the stability of DNA^38,47^ however the lyophilization process is also dehydrating. Studies have shown that DNA needs to maintain its hydration sphere to be stable^48,49^, therefore removal of this hydration sphere during the lyophilization process can lead to DNA damage. It has been reported that lyophilization without protective agents can lead to significant loss in transfection efficiency^37,39^ and therefore activity. Several studies have been carried out to study the impact of lyophilization on pDNA, however the impact of lyophilization on a linear covalently closed molecule like msDNA is currently unknown.

**Fig. 5:**
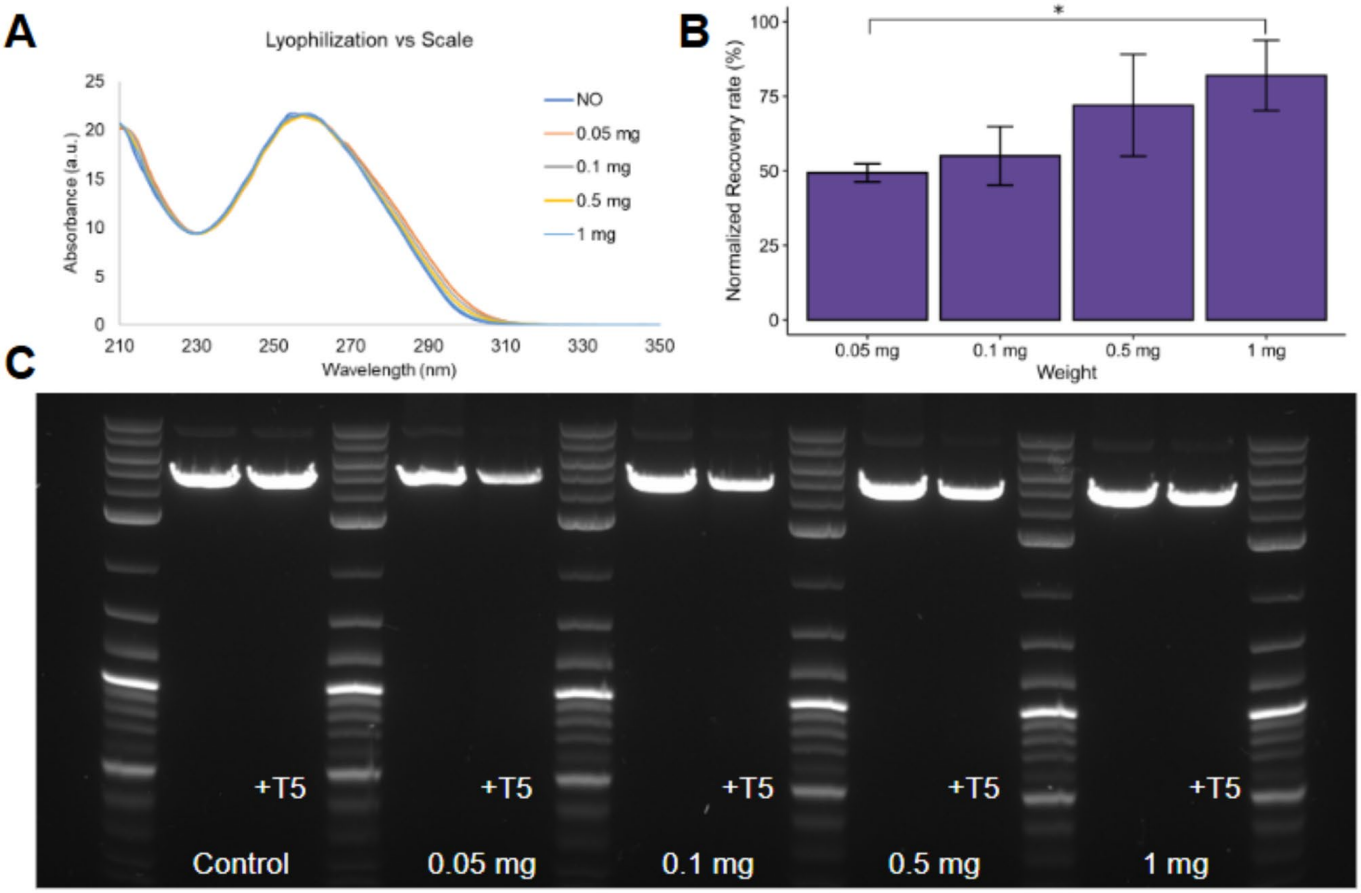
Stability assessment of msDNA (Construct A, 4.3 kb, 59% GC) to lyophilization at various scales in water. (**A**): UV-Vis spectra of the various DNA samples before and after lyophilization, with the NO (Control) sample representing the pre-lyophilization sample. The 0.05, 0.1, 0.5 and 1.0 mg indicate the different scales of lyophilization. (**B**) Quantitative analysis of the recovery following T5-exonuclease treatment observed in the agarose gel electrophoresis (AGE) gel. Bars show the average of three technical replicates, and error bars show one standard deviation. One-way ANOVA with TukeyHSD test: *p < 0.05 (Table S4). (**C**) AGE gel of the lyophilization samples treated with/without T5 exonuclease.

In our first tests (Fig. 5 and Figure S4), we conducted lyophilization of msDNA (Constructs A-F, Table 1) in water without any additives at the 0.05 mg (50 µg) scale. Our results indicate that msDNA without any additives is not very stable after lyophilization as it undergoes significant damage by T5-exonuclease after lyophilization (Fig. 5, Figure S4 and Table S4). As expected, the DNA damage upon lyophilization is dependent on the nature of the DNA construct, with the recovery rates varying between 10 and 90%. Larger DNA molecules undergo significantly more DNA damage than smaller DNA molecules, demonstrated with our larger construct E (10.3 kb) having only an 11% recovery, as opposed to the smaller construct F (1.6 kb) having a ~ 90% recovery. Additionally, A significant spectral shift (~ 5–10 nm) in the UV-Vis spectrum was observed for samples damaged during lyophilization without excipients (Figures S4A, S4B, S4D), whereas the relatively stable Construct A (Fig. 5A) show minimal changes. A shift in absorbance indicates potential denaturation and changes in DNA base stacking, as observed in samples undergoing lyophilization induced damage. This observation aligns with previous findings where plasmid DNA exhibited a hyperchromic shift, resulting in decreased transfection efficiency^39^. We hypothesize that lyophilization potentially denatures the DNA and causes changes in the DNA base stacking^37,38^, leading to the hyperchromic/red shift^37,38^. DNA can adopt many forms, with A-DNA, B-DNA, and Z-DNA relevant for biological activity. The B-form is the most stable in solution and is most found; however, DNA is driven into A-form and Z-form in dehydrating conditions^50^ like those during the lyophilization process.

Interestingly, we found that lyophilization stability improves at larger scales. At a 1 mg scale, recovery increased to ~ 85%, compared to only 50% at the 0.05 mg scale, likely due to entropy-driven stability under conditions where DNA crowding occurs^51^.

#### DNA form and lyophilization process

To test the role that various manufacturing processes play in the stability of DNA, we treated the DNA with two salt solutions that are typically used in the DNA manufacturing process^52–56^: (1) 0.3 M guanidine-HCl or (2) 3 M ammonium sulfate for a short period (1–3 h) before exchanging the samples into water. Following this treatment, samples were lyophilized, and subjected to stability tests.

**Fig. 6:**
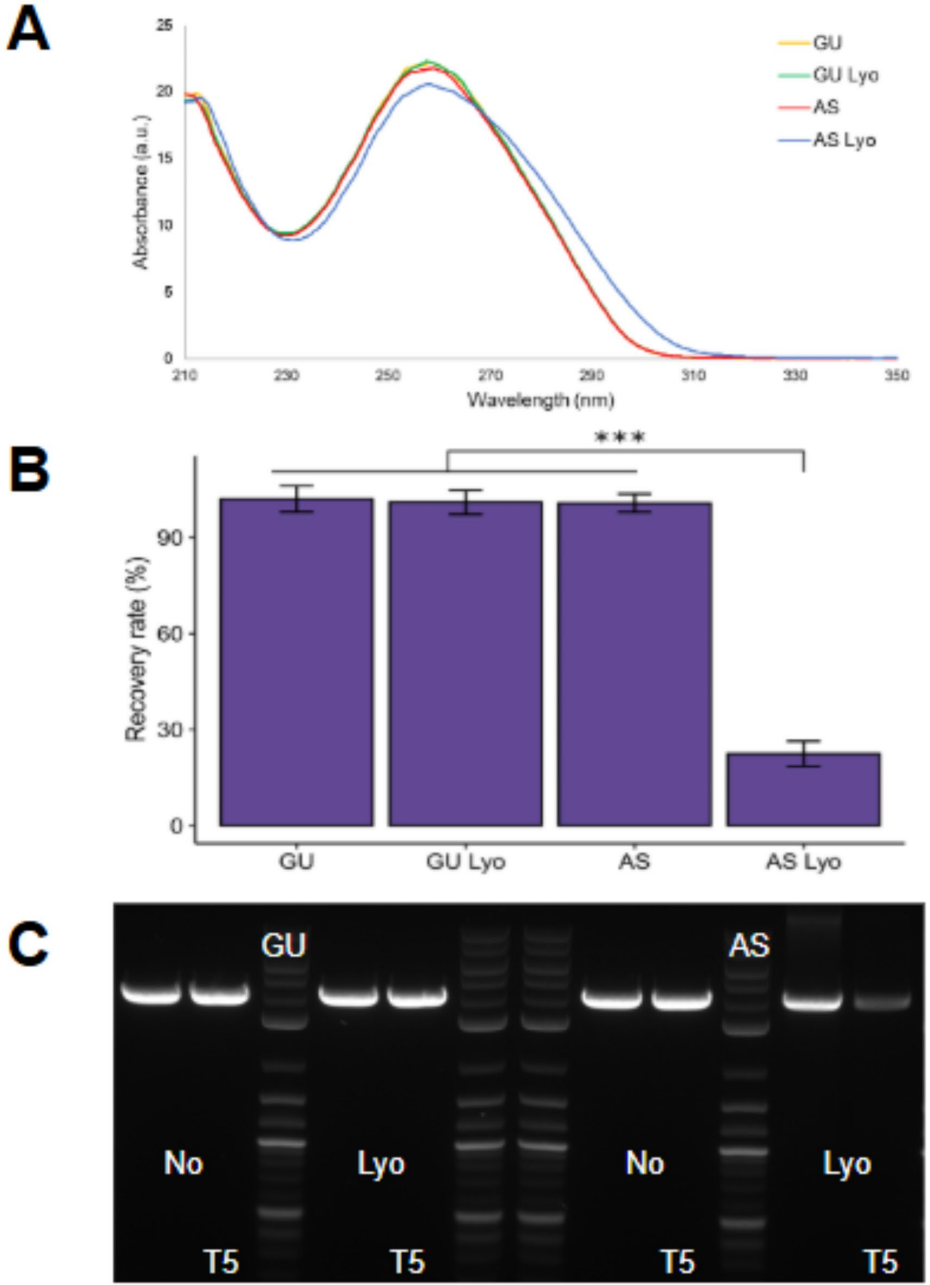
Interactions of msDNA (Construct G, 3.7 kb, 52% GC) with salts prior to lyophilization. (**A**): UV spectra of samples treated with Guanidine (GU) and Ammonium Sulfate (AS) treated samples before and after lyophilization (after lyophilization indicated by Lyo). The AS sample shows a strong hyperchromic shift post lyophilization. (**B**) Quantitative analysis of the recovery following T5-exonuclease treatment observed in the agarose gel electrophoresis (AGE) gel. Bars show the average of three technical replicates, and error bars show one standard deviation. One-way ANOVA with TukeyHSD test: ***p < 0.001 (Table S5). (**C**): AGE image of GU and AS samples treated with T5, assessing integrity after lyophilization.

Our results show that the sample obtained after treatment with guanidine-HCl (Fig. 6 and Table S5) does not show any significant damage after lyophilization with ~ 100% recovery and no shifts in the UV-Vis spectra. However, the samples treated with ammonium sulfate showed significant damage after lyophilization, as indicated by a large hyperchromic shift in the UV-Vis spectra (Fig. 6, AS Lyo) and poor recovery (only 25%) after treatment with T5-exonuclease. This is particularly important for non-viral gene therapy applications since ammonium sulfate is frequently used in downstream DNA purification, which may introduce variability in therapeutic efficacy.

The addition of guanidine-HCl improves the solubility of DNA in water (“salting-in” effect) and is expected to drive the DNA into the B-form^57^. The addition of ammonium sulfate would lower the DNA solubility (“salting-out” effect) and is expected to increase the proportion of the more compact DNA forms (ex: A-form and Z-form)^58^. We hypothesized that lyophilization from the more relaxed B-form would lead to a more stable lyophilized product. The presence of these alternative forms (A-form and Z-form) in varying amounts from the purification process might introduce some variability in the effectiveness of the therapy. Varying amounts of these alternative forms of DNA could lead to large differences in the size and polydispersity index (PDI) of the LNP-formulated DNA and, by extension, the DNA delivery efficiency and potency as API.

#### Improving DNA lyophilization by excipient addition

While, A-form, B-form, and Z-form DNA molecules can function biologically, stabilizing the DNA in one form via excipient addition prior to lyophilization is critical to reduce variability. A typical lyophilization process contains three steps: (1) freezing, (2) primary drying (sublimation), and (3) secondary drying (desorption)^59,60^. Each stage can introduce stress to DNA, potentially disrupting its structure. The addition of excipients, such as sugars, can mitigate these effects by promoting stable glass formation during freezing, preserving DNA’s extensive hydrogen-bonding network. Moreover, during drying, sugars can substitute for water molecules within the DNA helix, maintaining a pseudo-hydrated state that enhances structural integrity^37,38^.

Therefore, we evaluated the addition of two sugars (1) glucose, and (2) trehalose. Glucose is a monosaccharide reducing sugar while trehalose is a non-reducing disaccharide sugar that combines two glucose molecules connected by an α,α − 1,1-glycosidic bond. We selected a concentration of 10 mM of sugar for 1 mg/mL (~ 3.3 mM DNA bases) of DNA (~ 3-fold excess of sugar to DNA bases).

**Fig. 7:**
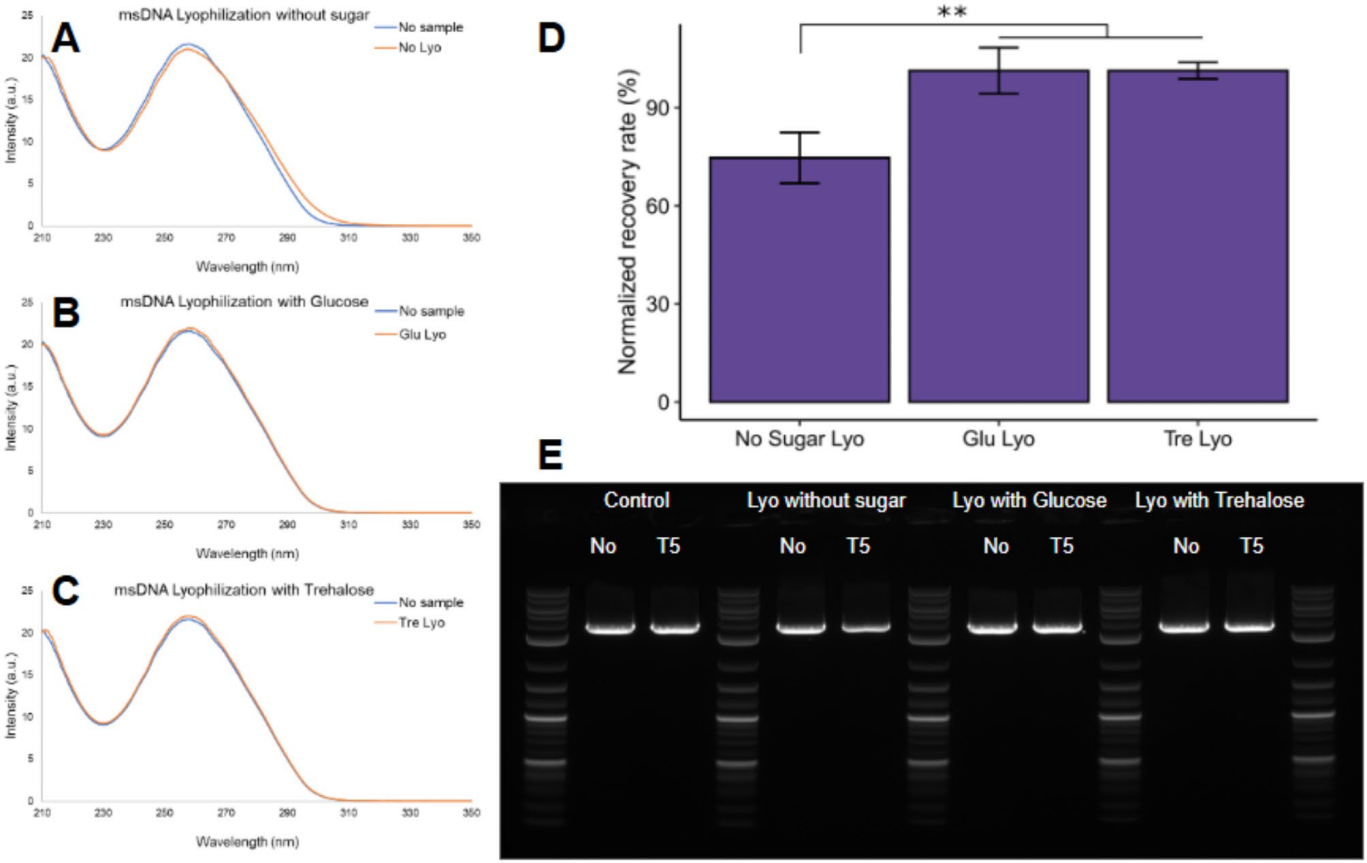
Role of excipient in the stability of msDNA (Construct G, 3.7 kb, 52% GC). (**A**, **B**, **C**): UV-vis spectra of the samples before (blue) and after (orange) lyophilization. (**D** and **E**): Quantitative analysis and AGE of the samples before and after lyophilization and their corresponding recovery rates after exposure to T5-exonuclease. The trehalose additive demonstrates the most effective protection against lyophilization-induced damage. Bars show the average of three technical replicates, and error bars show one standard deviation. One-way ANOVA with TukeyHSD test: **p < 0.005 (Table S6).

Both sugars significantly improved the stability of msDNA (Construct G) post-lyophilization, with recoveries of 94% and ~ 100%, respectively (Fig. 7 and Table S6), compared to 66–70% recovery in the absence of sugars. However, glucose, as a reducing sugar, poses a risk of forming covalent adducts with biomolecules, potentially compromising msDNA functionality. In contrast, trehalose offers additional protective benefits, including resistance to UV-induced damage and reactive oxygen species^47,61^. Consequently, we selected trehalose for further studies. The addition of 10 mM trehalose universally enhanced the stability of various msDNA constructs (Figure S5), which were chosen to represent a range of sizes and GC contents relevant to gene therapy applications, including non-viral vectors and viral vector production (e.g., AAV and lentivirus). These constructs incorporated specialized genetic elements—such as inverted terminal repeats (ITRs) for AAV, long terminal repeats (LTRs) for lentivirus, and long polyA tails for RNA manufacturing—whose stability can vary under stress.

Our results revealed that larger constructs (> 5 kb) were more prone to damage during lyophilization without excipients (Table S1, Figure S4). Trehalose supplementation significantly alleviated this vulnerability. For example, Construct E, which exhibited only 11% recovery without excipient, achieved ~ 100% recovery with trehalose (Figure S5), demonstrating a dramatic improvement in stability across all tested constructs.

Finally, we assessed whether trehalose addition during lyophilization affected the biological activity of the DNA cargo using an in-vitro potency assay. Transfection of msDNA samples (Construct A) before and after lyophilization into HEK293 cells revealed comparable transfection efficiencies and expression levels of GFP.

We further evaluated if lyophilization with 10 mM trehalose has any impact on the in vitro potency and expression in cell cultures. To perform this experiment, we transfected msDNA samples (Construct A) before and after lyophilization into HEK 293 and observed similar transfection efficiencies and expression of the reporter gene (GFP). Our results (Figure S6) indicate that lyophilization with trehalose does not adversely affect DNA biological functionality, suggesting that excipient addition can enhance the stability of lyophilized products without compromising their efficacy.

#### Long-term stability study of the lyophilized product

**Fig. 8:**
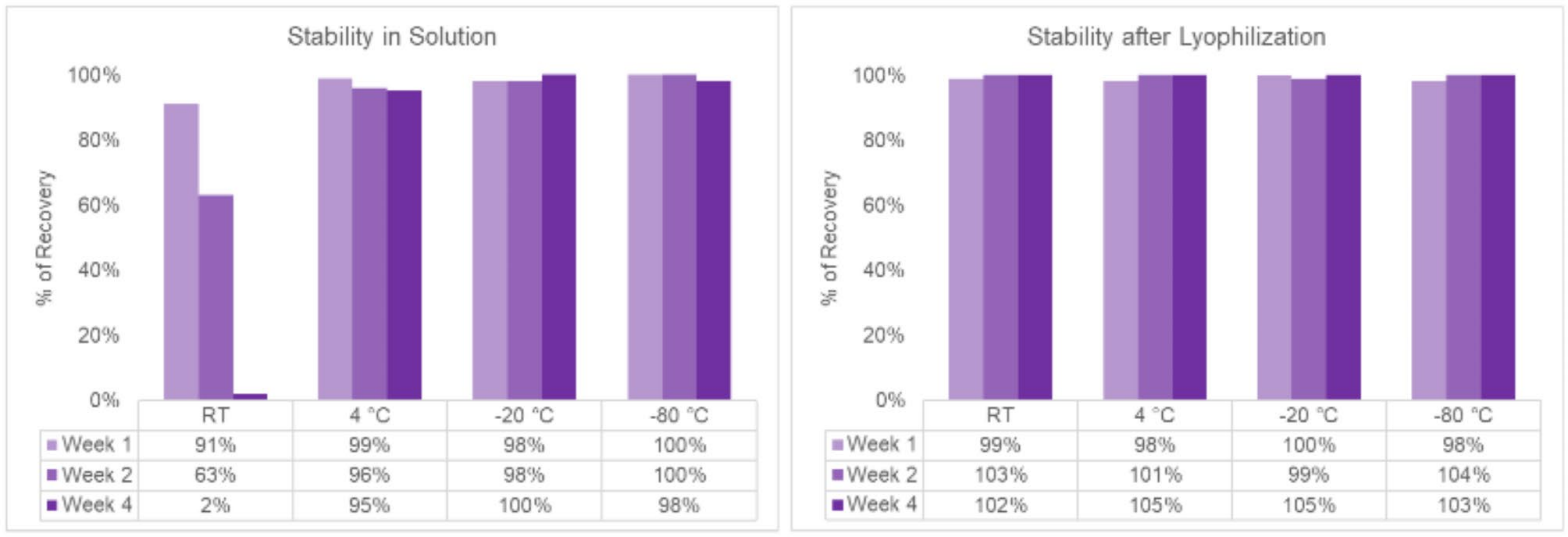
Long term stability of msDNA (Construct A, 4.3 kb, 59% GC) under different storage conditions. At low temperatures (4 °C, -20 °C and − 80 °C), msDNA remains stable in both solution (**A**) and lyophilized form (**B**). At room temperature (RT), msDNA is stable in-solution for approximately 1 week only, but it maintains stability in lyophilized form for at least 4 weeks. The raw data supporting this graph can be found in Figures S7-S9. Note: AGE analysis has an error around 5–10% therefore values near 95–105% are considered complete recovery or no loss.

To assess long-term stability, we stored the lyophilized msDNA sample (Construct A) with the optimized process—containing 10 mM trehalose—over extended periods. Samples (in solution or after lyophilization) were stored in the dark at various temperatures (room temperature [~ 23 °C], 4 °C, -20 °C or -80 °C) to prevent potential light-induced damage. As expected, samples stored frozen at -20 °C or -80 °C demonstrated minimal damage, both in solution and post-lyophilization. However, samples stored in solution at room temperature exhibited significant DNA damage after 4 weeks (with only ~ 2% survival), while the lyophilized samples remained completely stable without T5-exonuclease-induced damage (Fig. 8, Figure S7, Figure S8, and Figure S9). These results highlight that lyophilization in the presence of excipients can significantly enhance the long-term stability of msDNA at ambient temperatures, thereby mitigating the necessity for cold chain storage during transport.

## Discussion

This study provides a comprehensive evaluation of the stability of ministring DNA (msDNA), a novel bacterial-sequence-free linear covalently-closed (LCC) miniDNA vector, against both chemical and mechanical stresses. Our findings highlight msDNA’s unique structural advantages, which contribute to its enhanced stability and suggest its potential as a safer and more effective DNA vector in gene therapy applications. The successful optimization of lyophilization further supports msDNA’s long-term storage capabilities, enabling room-temperature preservation and transportation.

### Stability and structural advantages

msDNA stands out due to its linear, torsion-free configuration, eliminating the supercoiling typical of plasmid DNA. Supercoiled plasmid DNA helps condense the DNA in a crowded cellular environment and often requires unwinding for transcription and replication^23^. However, this supercoiling and unwinding can introduce further torsional forces that can lead to structural vulnerabilities^62^. In contrast, we hypothesize that msDNA’s linear form promotes a more relaxed conformation that resists destabilizing forces, including pH fluctuations and mechanical stress, such as sonication. The enhanced stability observed at pH 4, a critical condition for lipid nanoparticle (LNP) formulations, is particularly noteworthy. Our results demonstrate significant degradation of DNA at pH 3 but maintain the structural integrity of msDNA at pH 4, making it a promising candidate for LNP-based delivery systems^63,64^. Typically, LNP formulations, such as those employed in mRNA vaccines, incorporate ionizable lipids that exhibit positive charge at pH levels below their pKa and are uncharged near neutral pH levels^65^. For optimal encapsulation of nucleic acid molecules (whether mRNA or DNA) by LNPs, it is common practice to incubate DNA in low pH buffers (pH 3 or 4) before rapidly mixing with ionizable lipids. Our findings (Figure S2) reveal significant DNA damage at pH 3 following short incubation periods (~ 1 h), strongly recommending the avoidance of such acidic conditions in LNP formulation efforts. Low pH (pH 3) causes DNA damage including depurination^66^ that is not easily observed by agarose gel electrophoresis but makes the DNA susceptible to T5-exo activity. Depurination is also a leading cause of mutations^67^ and is poorly studied for nucleic acid molecules (RNA or DNA) undergoing the LNP encapsulation process. DNA damage by low pH could also introduce reactive open ends that heighten the risk of generating nicked DNA. These reactive ends are particularly concerning in therapeutic contexts, as they can lead to unregulated and random integration and insertional mutagenesis causing genomic instability—critical safety concerns for applications involving gene delivery and gene therapy. Conversely, msDNA exhibits resilience at pH 4, showing negligible degradation, making it a viable candidate for LNP applications. The structural stability of msDNA likely reduces variability in DNA-LNP formulations, thus yielding more consistent and effective non-viral gene delivery systems.

### Lyophilization and room-temperature storage

The need for cold chain storage in current genetic medicines, such as mRNA vaccines and adeno-associated virus (AAV) vectors, poses significant logistical challenges, particularly in low-resource settings^13,68^. The successful lyophilization of msDNA with trehalose as an excipient offers a compelling solution to this problem. The addition of 10 mM Trehalose not only stabilized msDNA during the freeze-drying process but also maintained the DNA’s functionality and integrity post-lyophilization. The ability to store msDNA at room temperature without degradation facilitates broader accessibility and distribution of gene therapies, particularly in regions with limited infrastructure. It is important to note that stability of DNA post-lyophilization will likely vary by construct (due to size and genetic elements), and future studies should assess each construct individually.

### Insights from DNA form and lyophilization

The processes employed in DNA manufacturing directly influence its stability during lyophilization. The transition of DNA between forms (B-form vs. A- or Z-form) prior to freezing significantly affects stability. Chaotropic agents like guanidine hydrochloride are known to stabilize DNA in its B-form, whereas kosmotropic agents such as ammonium sulfate can destabilize the DNA, shifting it to the less stable A or Z forms. Notably, ammonium sulfate, commonly utilized in downstream processing^53,54^, can adversely affect DNA stability even after its removal, suggesting that DNA does not easily revert to its most stable conformation. This indicates a persistent impact on stability, emphasizing the necessity of incorporating excipients to enhance stability. These insights are pivotal for designing processes that improve DNA product quality and stability, thereby advancing therapeutic gene therapy and genetic medicine.

### Implications for gene therapy and manufacturing

The structural integrity of msDNA contributes to its unique positioning as the only LCC miniDNA vector manufactured entirely in vivo in *E. coli *^23,25,26^. This approach leverages the endogenous *E. coli* mismatch repair (MMR) system^30^ to achieve high-fidelity DNA products. With the established scalability and regulatory compliance of *E. coli* driven production ^18,69,70^, there is potential for msDNA to serve as a benchmark standard for various applications— including non-viral gene delivery, viral vector manufacturing (e.g., AAV and lentivirus), and as a template for mRNA production. This positions msDNA as a potentially universal DNA vector, meeting both therapeutic and manufacturing needs.

### Future research directions

The structural integrity of msDNA is crucial for its performance, especially in the context of pH changes and sonication, which can adversely affect DNA functionality. Stable DNA cargoes with high integrity are essential for effective gene transfer and expression. Preserving DNA structure, particularly avoiding nicked ends, is critical to ensuring safety and minimizing the risks associated with unintended mutations, genomic instability, and potential oncogenic transformations. In the next phase of research, we will evaluate the impact of different ex vivo physical delivery techniques, such as ultrasound and electroporation, on the stability of msDNA. We will also compare the stability profiles and performance attributes of traditional plasmids versus closed double-stranded linear msDNA constructs. By assessing how different delivery methods influence DNA integrity, we aim to elucidate the advantages of msDNA in gene and cell therapy applications. Understanding these dynamics will be essential for optimizing the design of DNA vectors and enhancing the overall safety and efficacy of genetic medicines.

## Conclusion

In summary, this study shows that msDNA has enhanced stability to chemical and mechanical stress, likely due to its LCC topology compared to other DNA vectors used in gene therapy. Using lyophilization, we have developed a process that can store msDNA for at least four weeks at ambient temperatures, eliminating the need for refrigerated transport. msDNA is a safer option for the manufacturing of viral vectors and as an API due to its profound immuno-compatibility, achieved by eliminating immunogenic bacterial sequences. Additionally, its LCC conformation mitigates the risks of random integration and insertional mutagenesis, causing chromosomal disruption leading to cell death in the unlikely event of a potentially oncogenic integration. With its added advantages of *E. coli*-based manufacturing, scalability, cost-effectiveness, and enhanced fidelity, msDNA has the potential to be a novel, safe DNA-based standard for a wide range of gene therapy, gene editing, and DNA/RNA vaccine applications. msDNA can be utilized as, including starting materials or therapeutic ingredients to support different gene and cell therapy manufacturing, gene therapy, gene editing, and DNA/RNA vaccine modalities.

## Electronic supplementary material

Below is the link to the electronic supplementary material.

Supplementary Material 1

## Data Availability

Data is provided within the manuscript or supplementary information files. In case of any questions please contact – Siddarth Chandrasekaran (cyddarth@gmail.com) or Nafiseh Nafissi (nafiseh.nafissi@mediphage.ca) for any further information.
