# Supplementary material for: Ministring DNA (msDNA): a novel linear covalently-closed DNA with enhanced stability for gene and cell therapy applications
Source: Sci Rep. 2025 May 2;15:15420. doi: 10.1038/s41598-025-98730-5 (PMC12048660; doi:10.1038/s41598-025-98730-5)
Supplement: Supplementary file 1 — Supplementary Material 1 [file 41598_2025_98730_MOESM1_ESM.docx]

Supplementary Data:

Table S1: The stability of different msDNA^TM^ constructs to lyophilization.

The addition of trehalose effectively stabilizes the DNA and prevents any lyophilization damage.

| **msDNA**^TM^ **Construct** | **Lyophilization stability/recovery** | |
| --- | --- | --- |
|  | No excipient | 10mM Trehalose |
| A | 48% | 100% |
| B | 46 % | 99 % |
| C | 40 % | 102 % |
| D | 85 % | 99 % |
| E | 11 % | 104 % |
| F | 90 % | 101 % |
| G | 66 % | 101 % |

*Table S2: T-test results comparing the stability of msDNA^TM^ and pDNA to weak and strong sonication. More details are presented in Figure 3.*

| **Sonication** | **Time** | **pValue** | **Sig** |
| --- | --- | --- | --- |
| **Weak** | 10s | 0.102135 | NS |
| **Weak** | 30s | 0.000525 | *** |
| **Weak** | 1_min | 0.073028 | NS |
| **Weak** | 10_min | 0.373901 | NS |
| **Strong** | 5s | 0.139619 | NS |
| **Strong** | 10s | 0.026386 | * |
| **Strong** | 30s | 0.373901 | NS |
| **Strong** | 1_min | NA | ND |

*Table S3: T-test results comparing the stability of msDNA^TM^ and pDNA to chemical stress (pH). More details are presented in Figure S2 (1 hr) and Figure 4 (O/N).*

| **pH** | **Time** | **pValue** | **Sig** |
| --- | --- | --- | --- |
| pH_3 | 1_hr | 0.049072 | * |
| pH_4 | 1_hr | 0.961496 | NS |
| pH_5 | 1_hr | 0.656934 | NS |
| pH_7 | 1_hr | 0.086898 | NS |
| pH_9 | 1_hr | 0.479076 | NS |
| pH_10.5 | 1_hr | 0.012345 | * |
| pH_3 | O/N | NA | ND |
| pH_4 | O/N | 0.022583 | * |
| pH_5 | O/N | 0.060829 | NS |
| pH_7 | O/N | 0.215416 | NS |
| pH_9 | O/N | 0.539753 | NS |
| pH_10.5 | O/N | 0.058885 | NS |

*Table S4: Results from the one-way ANNOVA followed by post hoc Tukey test for the lyophilization experiments at different scales presented in Figures 5.*

| **Comparisons** | **Adjusted_pValue** |
| --- | --- |
| 0.10_mg_Lyo-0.05_mg_Lyo | 0.929602 |
| 0.50_mg_Lyo-0.05_mg_Lyo | 0.155421 |
| 1.00_mg_Lyo-0.05_mg_Lyo | 0.035254 |
| 0.50_mg_Lyo-0.10_mg_Lyo | 0.341185 |
| 1.00_mg_Lyo-0.10_mg_Lyo | 0.081856 |
| 1.00_mg_Lyo-0.50_mg_Lyo | 0.723253 |

*Table S5: Results from the one-way ANNOVA followed by post hoc Tukey test for the lyophilization experiments comparing different manufacturing processes presented in Figures 6.*

| **Comparison** | **Adjusted_pValue** |
| --- | --- |
| AS_NO-AS_Lyo | 3.22E-12 |
| GU_Lyo-AS_Lyo | 3.13E-12 |
| GU_No-AS_Lyo | 2.84E-12 |
| GU_Lyo-AS_NO | 0.999661145 |
| GU_No-AS_NO | 0.961756361 |
| GU_No-GU_Lyo | 0.979664039 |

*Table S6: Results from the one-way ANNOVA followed by post hoc Tukey test for the lyophilization experiments comparing various excipients presented in Figures 7.*

| **Comparison** | **Adjusted_pValue** |
| --- | --- |
| NoSugar_Lyo-Glu_Lyo | 0.004606011 |
| Tre_Lyo-Glu_Lyo | 1 |
| Tre_Lyo-NoSugar_Lyo | 0.004606011 |


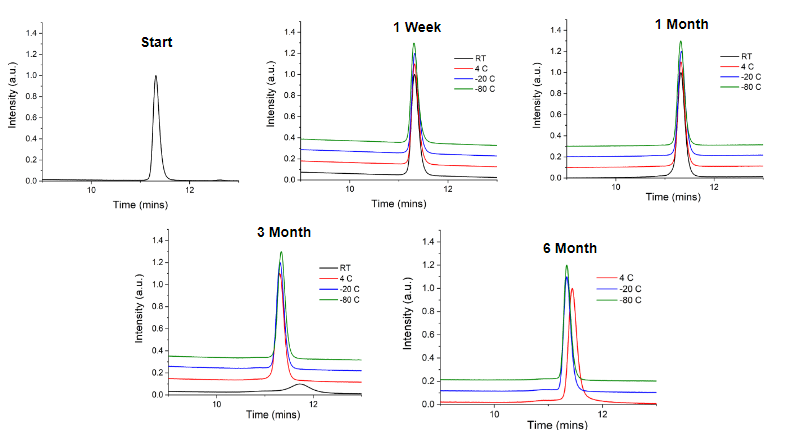


Figure S1: The stability assessment of msDNA^TM^ (Construct M) studied by UHPLC.

The plots show the UV absorbance at 260 nm against the elution time from our QC column at each of the following temperatures (Room Temperature ~23-25 ℃, 4 ℃, -20 ℃ and -80 ℃). Peak reductions and shifts indicate the degradation of our msDNA^TM^ product.


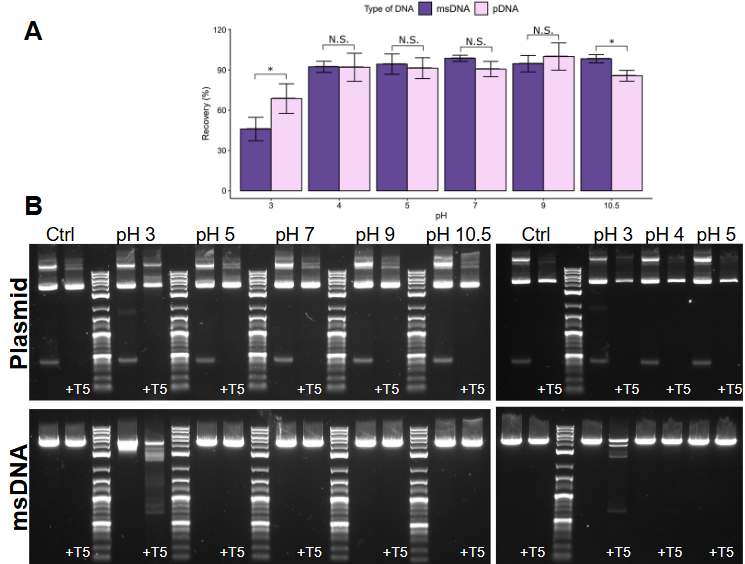


Figure S2: Stability assessment of msDNA™ and pDNA under chemical (pH) stress after 1-Hour Incubation. Plasmid DNA (pDNA) and ministring DNA (msDNA™) were incubated for 1 hour across a range of pH conditions to evaluate their stability. (A) Quantitative analysis of agarose gel electrophoresis (AGE) comparing the stability of msDNA™ and pDNA. Band intensities were quantified, with bars representing the mean of three biological replicates and error bars indicating one standard deviation. T-test: *p<0.05, , the statistics data is summarized in Table S3. (B): Agarose gel electrophoresis (AGE) of pDNA and msDNA™ under varying pH conditions. Both msDNA™ and pDNA remain stable between pH 5 and 10.5 but exhibit degradation below pH 4. For pDNA, only the supercoiled fraction (the predominant band between 4–5 kb) was quantified, as other isoforms (e.g., nicked, open circular, and linearized forms) are fully processed by T5 exonuclease. Stability over longer incubation periods is presented in Figure 4.


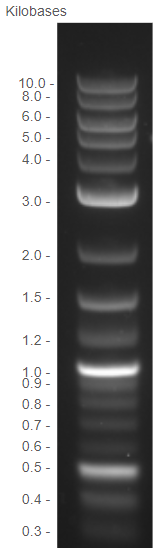


Figure S3: NEB 1 kb Plus DNA Ladder with different DNA sizes indicated on the lefthand side of the image (kb).

The bright bands at 3 kb, 1 kb and 0.5 kb are easily recognized. This ladder was used for all the agarose gels run in this paper.


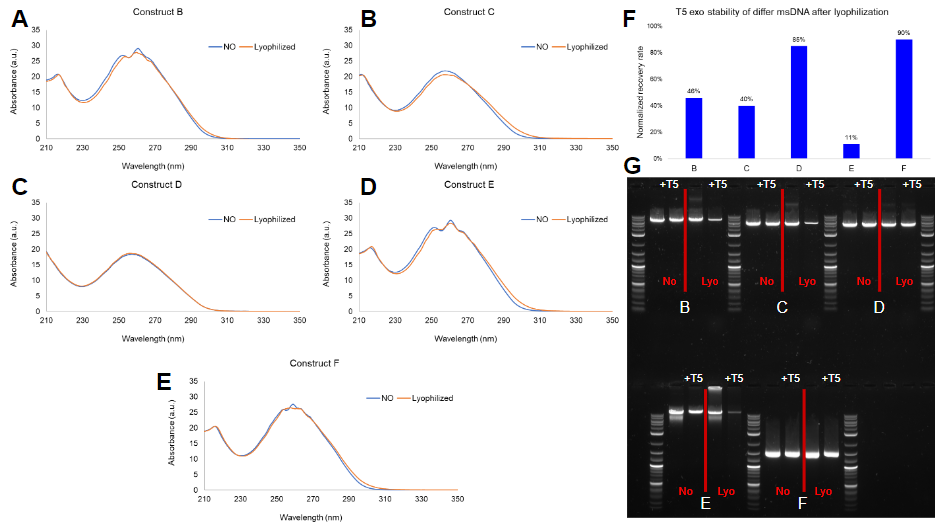


Figure S4: Impact of lyophilization on stability of various msDNA™ constructs (B, C, D, E, and F). Details of the constructs, including size, GC content, and genetic elements, are provided in Table 1. (A–E) UV-Vis spectra of msDNA™ constructs (B, C, D, E, and F, respectively) before lyophilization (labeled "NO") and after lyophilization (labeled "Lyophilized"). Spectra were recorded to assess changes in absorbance profiles post-treatment. (F–G) Quantitative analysis and agarose gel electrophoresis (AGE) of the constructs before (NO) and after (Lyo) lyophilization, following treatment with T5 exonuclease (T5-exo). Panel G shows AGE results, with band intensities quantified in Panel F as a bar graph. Overall, the different constructs vary between 10-90% for their stability to T5-exonuclease after lyophilization.


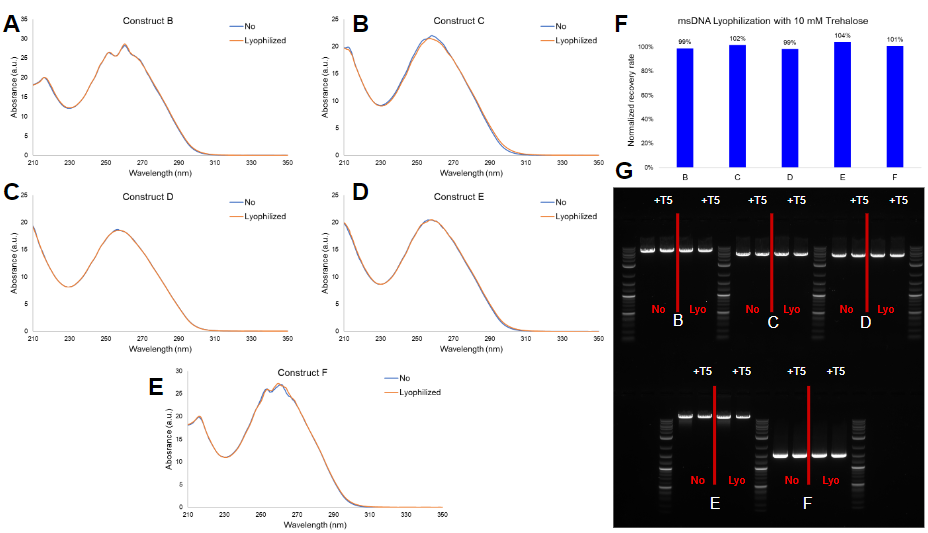


Figure S5: Impact of Lyophilization with excipient on stability of msDNA™ Constructs (B, C, D, E, and F). Various msDNA™ constructs (B, C, D, E, and F) were lyophilized using an optimized process with trehalose as an excipient. Details of the constructs, including size, GC content, and genetic elements, are provided in Table 1. (A–E) UV-Vis spectra of msDNA™ constructs (B, C, D, E, and F, respectively) before lyophilization (labeled "NO") and after lyophilization (labeled "Lyophilized"). Spectra were recorded to assess changes in absorbance profiles post-treatment. (F–G) Quantitative analysis and agarose gel electrophoresis (AGE) of the constructs before (NO) and after (Lyo) lyophilization, following treatment with T5 exonuclease (T5-exo). Panel G shows AGE results, with band intensities quantified in Panel F as a bar graph. . This compares with Figure S4 in which the same constructs were tested without excipient addition. The addition of trehalose significantly stabilizes the DNA during the lyophilization process and prevents any significant damage for all the constructs assessed.


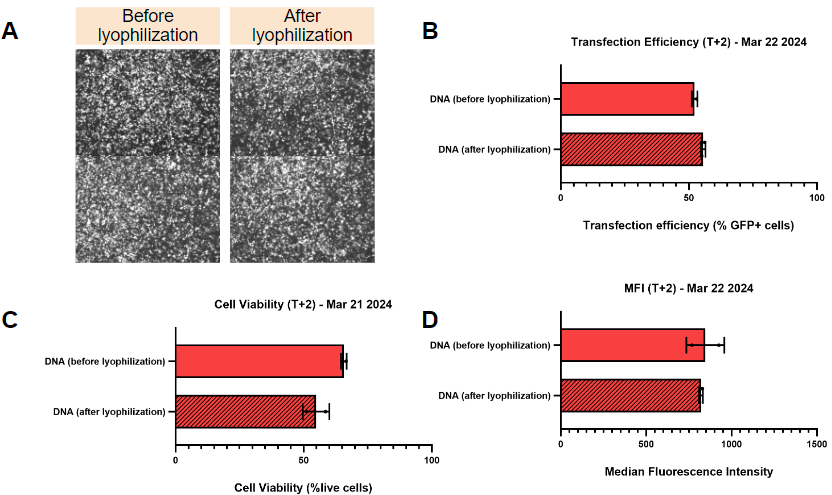


Figure S6. Expression of reporter (GFP) from msDNA^TM^ (**Construct A**) before and after lyophilization.

(A)The raw GFP images (GFP channel) of the HEK-293 cells transfected with Construct A along with flow cytometry analysis (B-D) of the cells after 2 days. Lyophilization with excipient has no impact on the GFP+ cells (transfection efficiency(B) or MFI (D)). The cell viabilities (C) are similar and within the error range for the experiment.


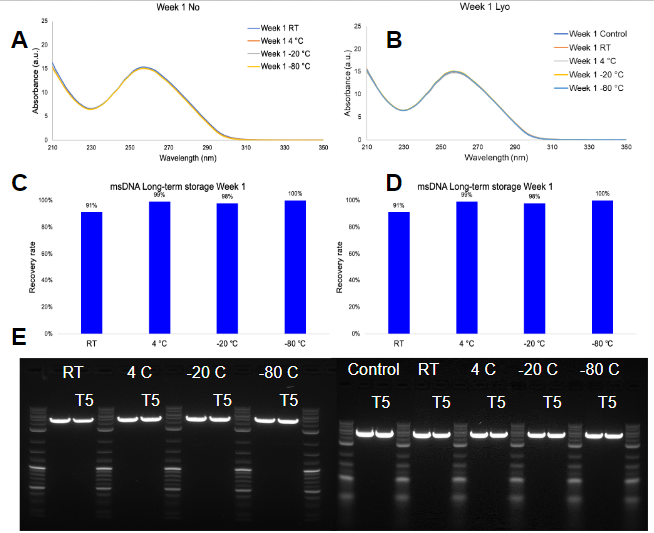


Figure S7: Long-term stability of msDNA™ Construct A – raw data from Week 1. This figure presents raw data from Week 1 of a long-term stability study of msDNA™ Construct A following lyophilization with excipient (trehalose). The details about construct A are provided in Table 1. (A–B) UV-Vis spectra of Construct A before lyophilization (labeled "NO") and after lyophilization (labeled "Lyo"), respectively, to assess absorbance profile changes post-treatment.


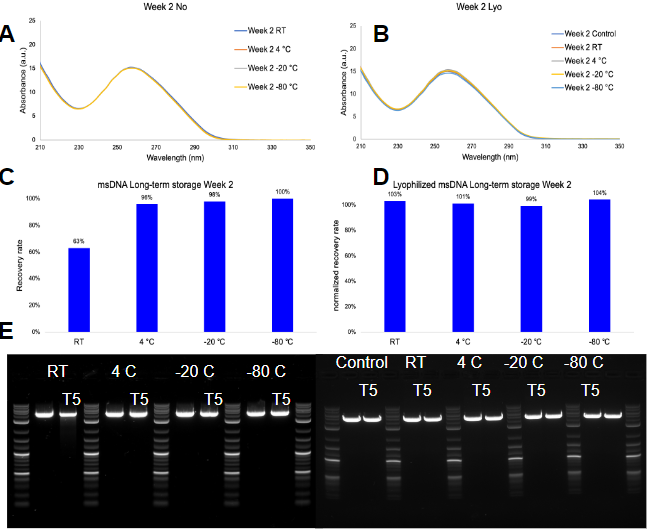


Figure S8: Long term stability of msDNA^TM^ (**Construct A**) study - Raw data from Week 2.

(A-B): UV-Vis before (NO) and after (Lyo) lyophilization. (C-D): Quantitative analysis of the AGE (E) for the samples stored at different temperatures and their stability to T5-exo.


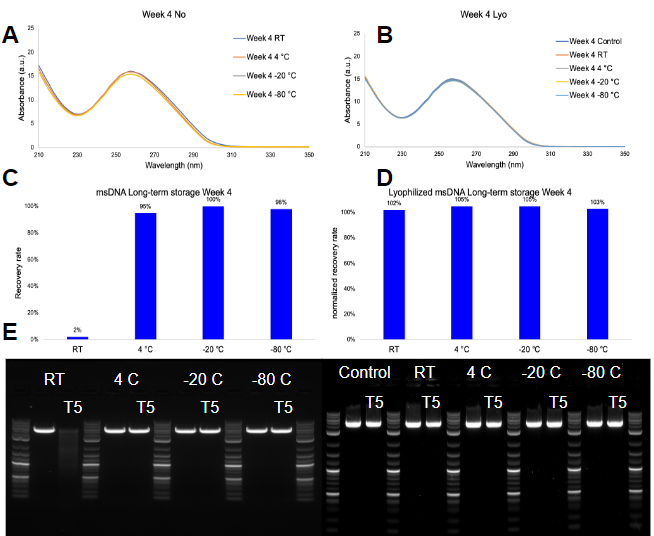


Figure S9: Long term stability of msDNA^TM^ (**Construct A**) study - Raw data from Week 4.

(A-B): UV-Vis before (NO) and after (Lyo) lyophilization. (C-D): Quantitative analysis of the AGE (E) for the samples stored at different temperatures and their stability to T5-exo.
